# Supplementary material for: Are carbon emissions trading and green financial instruments synergistic? -Comprehensive quantitative research based on content analysis
Source: PLoS One. 2024 Mar 7;19(3):e0298601. doi: 10.1371/journal.pone.0298601 (PMC10919601; doi:10.1371/journal.pone.0298601)
Supplement: S1 Data — (ZIP) [file pone.0298601.s001.zip › Data/地级市Z_F工作报告.zip!/╜¡╬≈/╝¬░▓╩╨/└┤╫╘╣1⁄2╓┌║┼í╛┬φ┐╦╩2╛▌═°í┐_2016.docx]

各位代表：

　　现在，我代表市人民政府向大会报告工作，请予审议，并请各位市政协委员和列席会议的同志提出意见。

　　一、2015年工作和“十二五”经济社会发展回顾

　　2015年是“十二五”的收官之年，也是我们适应新常态、夺取新胜利的重要一年。在省委、省政府和市委的坚强领导下，全市上下认真贯彻习近平总书记系列重要讲话特别是对江西工作“一个希望、三个着力”①重要指示精神，全面落实省委“十六字方针”，改革创新、攻坚克难，经济发展呈现逆势上扬、提速升级的良好态势，发展的坚韧性、协调性和惠民性不断增强，迈出了建设开放繁荣秀美幸福新吉安的坚实步伐。全市实现生产总值1328.5亿元、增长9.6%，财政总收入220亿元、增长12.7%，固定资产投资1487亿元、增长17.1%，社会消费品零售总额396.5亿元、增长12.8%，出口总额42.5亿美元、增长5.6%,城镇居民人均可支配收入27078元、增长9.2%，农村居民人均可支配收入10355元、增长11.8%，多项主要经济指标增幅居全省前列。

　　一年来，我们主要做了以下工作：

　　（一）综合施策稳增长，经济发展逆势上扬。面对持续加大的经济下行压力，把稳增长摆在首要位置，保持定力、精准发力。狠抓实体帮扶，及早制定帮扶企业“两个行动计划”，出台“稳增长32条”，帮助企业融资近50亿元、招工3.5万人，一批企业增资扩股，11家企业在区域股权交易中心成功挂牌，全市规模以上工业企业新增132户、达1095户，规模工业增加值730亿元、增长9.8%。狠抓投资融资，全市400个重点项目扎实推进，峡江水利枢纽9台机组全部并网发电，新干航电枢纽开工建设，金樟大道、庐陵大桥基本建成，新井冈山大桥即将合龙；全市争取地方政府债券49.5亿元，中心城区棚改项目国开行第一期31.4亿元专项贷款成功获批，赣西堤项目获中信银行20亿元无抵押贷款。狠抓招商引资，积极参加全国知名民企助推江西发展升级大会、赣港会、赣台会、华赣会等重大活动，先后举办各类专题招商活动73场次，全市引进内资518.8亿元、增长15.9%，实际利用外资8.83亿美元、增长12.4%；对台招商再创佳绩，新引进台资企业33户、台资2.65亿美元，均列全省第一。

　　（二）加大力度调结构，质量效益同步提高。在稳增长的同时，把调结构作为主攻方向，加快产业升级、区域升级。推动工业加快集聚，全市工业用电量增长7.3%，规模工业主营业务收入3000亿元，其中战略性新兴产业1145亿元，占规模工业比重38.2%、提高1个百分点；电子信息产业突破600亿元、增长13.2%，绿色食品产业突破300亿元、增长10.5%。加快壮大富民产业，粮食总产82亿斤，粮食收购量42亿斤、居全省第一；新增六大富民产业面积40万亩，其中井冈蜜柚新增9万亩，跻身全省三大果业品牌，遂川狗牯脑茶名列全省“四绿一红”茶品牌之首；“1+13”现代农业示范园全部达标挂牌，核心区总面积达3.58万亩。促进服务业扩量提质，服务业占GDP比重提高1.6个百分点，实现税收98亿元，占财政总收入的44.5%；新增吉州窑和燕坊古村2个国家4A级景区，井冈山旅游回暖升温，武功山和青原区旅游知名度不断提升，赣江水上旅游成功破题；金鑫未来港等一批高端城市综合体建成投用；农信社改制和村镇银行在全省率先实现县域全覆盖，金融机构贷款余额1055亿元，增长20.4%、居全省第二，金融业税收突破10亿元；电子商务交易额突破200亿元、连续两年翻番，快递业务量增速居全省第一。推进区域发展升级，经开区实现主营业务收入520亿元，全省排名前移1位；吉泰走廊“四化”协调发展示范区规划获国家批复，走廊实现生产总值697亿元，占全市比重达52%、提高0.8个百分点；新增吉水绿色食品、永丰碳酸钙2个县域百亿产业集群，新增永新、万安和峡江3个县财政总收入过10亿元，总数达11个，吉安县过25亿元。经开区“集聚度”、吉泰走廊“贡献度”和县域经济“均衡度”进一步提升。

　　（三）蹄疾步稳抓改革，内生动力不断增强。针对制约发展的体制机制障碍，着力推进一批重大改革，将改革红利转化为发展新动能。“一廊四区”②改革继续深化，工业园区体制改革焕发活力，中心城区城管体制改革取得突破，农村土地确权登记颁证基本完成，国有林场改革通过国家验收并在全国现场会上作经验交流，扩权强镇试点继续扩面并在全省推广，不动产登记制度有序实施，户籍制度改革深入推进。15项重点突破改革成效显著，财政专项资金竞争性分配走在全省前列。事业单位改革成功破题，市农科所和水利设计院改革稳步推进。政府综合改革和自身建设取得新成效，政府系统学法和法律顾问制度不断完善，领导干部违规插手干预工程问题专项治理深入推进，法治政府、廉洁政府建设呈现新气象；机构改革基本到位，简政放权不断深化，工程项目并联审批试运行，在全省率先取消非行政许可，“三单一网”③建设基本完成；国地税联合办税实现全覆盖；“三证合一”④商事登记制度全面推行，有力促进了“大众创业、万众创新”，全市新增私营企业和个体工商户分别增长26.1%、16.4%。

　　（四）坚定不移优生态，城乡品质持续提升。坚持以生态为底色、以文化显特色，着力促进生态建养与城乡建设协同并进。“两带一区”⑤建设顺利开局，新增一批郊野公园。“三改三清”⑥攻坚行动首战告捷。“三条红线”⑦基本划定，“十二大行动”⑧全面展开。农业面源污染防治有效加强，水库退养基本完成。在全省率先启动县市区空气质量自动监测站建设。全市新增省级生态乡镇7个，安福县跻身全省第一批生态文明先行示范县，经开区列入国家循环化改造示范试点，吉安获批国家循环经济示范城市。注重把生态和文化元素融入城乡建设，中心城区儒林里特色古街、庐陵人文谷和白鹭洲书院公园改造等项目扎实推进，全市城镇化率提高1.7个百分点、达46.35%。高标准规划建设88个中心村，完成872个村点建设，农村清洁工程和污水专项治理初见成效，一批新的美丽乡村示范带竞相呈现。全市主要河流水质保持在国家Ⅲ类标准以上，空气优良率达95%以上，环境质量居全国前列，吉安成为全省唯一全境入选“全国生态保护与建设示范区”的设区市。

　　（五）统筹兼顾促协调，社会事业加快发展。围绕“五位一体”总体布局，大力发展科教文卫事业，推动经济社会协调发展。创新驱动继续增强，新增博士后工作站4家、省级院士工作站2家、省级产业技术创新战略联盟2个、省级企业技术中心和研究中心10家，高新技术企业新增31户、总数达101户，高新技术产业增加值占规模工业的36.2%、提高8.9个百分点。教育事业加快发展，全市补充教师1679人，新建乡镇中心幼儿园28所，恢复农村教学点45个，高考成绩稳中有升，吉安职业技术学院办学规模和水平同步提升，职教联盟成功组建。卫生服务水平不断提升，上海市东方医院吉安医院建成开业，县级公立医院改革全面推开，大病保险实现全覆盖，市医疗急救指挥中心建成运行。文化体育事业繁荣发展，省级庐陵文化生态保护实验区正式获批，全国跳伞锦标赛、全省第二届畲族文化艺术节在吉安成功举办。

　　（六）全力以赴惠民生，共享发展取得实效。始终坚持把增进群众福祉作为最大责任，全市民生支出285亿元，占一般公共预算支出达79.3%。60件民生实事全面完成。新增城镇就业5.5万人、转移农村劳动力7.3万人。建成保障性住房16460套、棚户区改造5121套。居民医保、养老保险、城乡低保和社会救助扩面提标，第11次提高企业退休人员基本养老金标准。积极对接苏区振兴政策，争取中央和省预算内投资项目617个，资金21.6亿元、增长11.4%，农村公路、安全饮水、电力供应等基础设施普遍改善。大力推进精准扶贫，全市共投入扶贫资金25亿元，改造农村危旧土坯房64380户，减贫10万人，全省精准扶贫攻坚现场推进会在吉安召开。居民消费价格总水平上升1.5%，涨幅控制在范围之内。法治吉安、平安吉安建设扎实推进，安全生产和食品药品安全形势总体平稳。同时，国家安全、国防动员、拥军优属、人民防空、防震减灾工作进一步加强；统计、供销、公积金、方志、档案、民族宗教、外事侨务工作取得新成绩；妇女儿童、社会慈善、红十字会、残疾人及老龄事业实现新发展；气象、水文、海事、盐务、石油、机场、海关、检验检疫、高速路政、邮政通信等中央和省属单位服务地方发展作出了新贡献。

　　需要向代表报告的是，随着我国经济发展进入新常态，“十二五”后期增速普遍放缓，吉安与全国全省一样，面临前所未有的经济下行压力。2015年，全市社会消费品零售总额、固定资产投资、规模工业增加值实际增速，分别低于年初预期目标的0.7、1.4和2.2个百分点。对照“十二五”规划目标，全市固定资产投资、社会消费品零售总额增速分别低0.8和3.7个百分点。但总体看，我市“十二五”规划目标任务基本完成，全市生产总值年均增长11%，迈上1000亿元台阶，财政总收入突破200亿元，规模工业增加值三年翻番,固定资产投资和实际利用外资五年翻番，外贸出口五年翻两番。“十二五”时期成为我市经济发展速度最快、城乡面貌变化最大、人民群众获得感最多的时期之一，吉安站到了新的更高起点上。

　　———这五年，是深入推进“四化同步互动、三产共生演进”，产业结构不断优化的五年。工业主导地位全面凸显，传统产业和战略性新兴产业加快融合，电子信息产业跨越发展，主营业务收入近占全省“半壁江山”，跻身国家电子信息高新技术产业化基地、国家新型工业化电子信息产业示范基地。农业基础地位不断巩固，粮食生产连年丰收，被评为全国粮食生产先进市；井冈蜜柚产业快速壮大，种植面积达35万亩；现代农业示范园从无到有、从小到大，成为培训农民、培育产业和农业开放的主战场。服务业扩量提质，旅游休闲、商贸物流、电子商务、金融保险等业态蓬勃发展，服务业占GDP比重提高4.4个百分点，三次产业比重由19.8:50.5:29.7优化为16.4:49.5:34.1。

　　———这五年，是坚定不移“昂起龙头、挺起脊梁、立起支点”，区域经济协调发展的五年。经开区扩容提质，综合实力在全省工业园区排名前移5位，成功获批国家级出口加工区，通讯终端设备成为全市首个百亿产业集群，龙头带动作用不断增强。打造吉泰走廊重要增长带上升为省级和国家战略，19个重大平台获国家批复，吉安高新区升格为国家级高新区，走廊电子信息产业主营业务收入占全市的85%以上，“一路五桥”⑨相继建成，外环通道全面打通，“一核两翼三带”大吉安城镇群由概念变成雏形，生态红线管控在全省率先实行，“绿色空间相隔、快速通道相连、基础设施相接、改革创新先行、产业聚焦集聚、公共服务共享”的大格局基本形成，支撑带动作用明显增强。县域经济竞相发展，一批县域百亿产业集群加快形成，综合实力普遍增强。

　　———这五年，是加快建设“文化庐陵、山水吉安”，城乡面貌深刻变化的五年。以人为核心的新型城镇化进程不断加快，五年城镇化率提高8.7个百分点，全市城镇建成区面积增加43.7%，中心城区面积增加75.5%、达75平方公里，完成无物业管理老旧小区“五位一体”综合改造280多个。绿色生态优势加快提升，全市植树造林225万亩，新增国家湿地公园2个，庐陵文化生态园、古后河绿廊、螺湖湾湿地公园等一批精品工程相继建成，“精品公园+生态绿廊+郊野公园”三级梯度生态格局全面形成，吉安先后荣获全国绿化模范城市、国家森林城市、国家卫生城市、江西省首届生态宜居城市、全省文明城市、全国文明城市提名城市。庐陵风格美丽乡村建设呈现“全域规划、带状推进”的良好态势，累计投入43亿元，完成美丽乡村点建设4853个，“五美”内涵与“八不八多”理念深入人心，50多个镇村被评为全省历史文化名镇名村，100多万农民走上平坦路、喝上干净水，农村人居环境大为改善。

　　———这五年，是全面深化改革开放，动力活力竞相迸发的五年。深化改革导向鲜明、主线突出，一批重大改革破难而进、落地见效。工业园区体制改革纵深推进，实体化、市场化水平不断提升；城市建设与管理体制探索创新，庐陵新区成功组建并步入发展“快车道”；投融资体制机制障碍有效突破，七大政府投融资平台组建运营，交通、华夏、浦发等一批全国性商业银行落户吉安；七大系统非工口国企改革全面完成；社会事业创新发展，探索出合作办学、办医、办文化新模式。大开放主战略深入实施，实现由引企业向引产业转变，由工业开放向农业、服务业全领域开放转变。全市累计引进内资1981亿元，是“十一五”的3倍；实际利用外资38.6亿美元，是“十一五”的2.4倍，其中引进台资15亿美元，形成了台商集聚的“吉安现象”。

　　———这五年，是大力加强基础设施建设，区位综合优势不断提升的五年。高速公路通车里程突破600公里，五年净增260多公里，实现“县县通高速”。铁路运营里程超过450公里，高铁项目开工建设。井冈山机场旅客年吞吐量突破50万人次。新建和改造国省道430公里、农村公路9000公里，所有行政村通水泥路。全市电力装机容量新增60万千瓦，总量达358万千瓦。完成水库除险加固1038座，新建高标准农田65.8万亩，新增和恢复灌溉面积50万亩。

　　———这五年，是加快全面小康进程，人民生活水平显著提高的五年。教育事业大投入、大发展，仅中心城区就投入20多亿元，新建、改扩建学校15所，吉安一中、白鹭洲中学新校区建成投用，利用原市委大院扩建吉师附小，高职教育实现零突破。医疗卫生体系不断完善，每千人病床数由2.9张增加到4张，上海市东方医院吉安医院开创“特区带老区”合作办医新模式，医疗“空白村”全面消除。文化建设卓有成效，市文化艺术中心、工人文化宫、妇女儿童活动中心、城市展示馆、博物馆等一批公建项目竣工投用，“三馆一站”⑩实现县域全覆盖，“崇文、正气、开放、图强”的吉安城市精神充分彰显，文化大戏《记忆庐陵》走出国门、亮相世界。城乡居民养老保险、低保医保实现全覆盖。五年累计开工建设保障性住房和棚户区改造12.6万套。特别是全面打响新一轮精准扶贫攻坚战，六大扶贫举措深入实施，全市减少贫困人口32.6万人。城乡居民人均可支配收入分别是“十一五”末的1.74倍和1.86倍。公众安全感位居全省前列，连续三届荣获全省平安杯。

　　成绩来之不易，经验弥足珍贵。“十二五”时期吉安之所以能取得这些进步，关键是在市委的坚强领导下，全市上下展开一场“静悄悄的急行军”，以坚韧的政治定力、战略定力和实干定力，走上了一条革命老区振兴发展的新路子。五年来，我们始终坚持发展第一要务，实施四化同步互动、三产共生演进，主攻“园区、城区、景区和农业示范园”四大主战场，形成多元推进、多点发力的生动局面；始终坚持大开放主战略，全面深化改革，着力创新体制、放活基层，激发经济社会发展活力；始终坚持加快发展和负责任发展，自觉践行统筹、集约、绿色发展理念，构筑吉安发展新优势；始终坚持以人为本、民生为重，在城市，既拓展建设新城区又改造提升老城区，在农村，开辟美丽乡村和扶贫攻坚“两个主战场”，在社会领域，大力发展民生事业。这“四个始终坚持”既是五年来取得突出成绩的宝贵经验，更是今后推动吉安发展升级的重要遵循。

　　各位代表，我们深知，吉安的每一步发展，都得益于省委、省政府和市委的正确领导，得益于市人大、市政协和社会各界的鼎力支持，得益于全市人民的共同努力。在此，我代表市人民政府，向广大干部群众，向历任老领导、离退休老同志，向各民主党派、工商联、无党派人士和社会团体，向驻吉人民解放军、武警官兵和公安消防干警，向所有关心和支持吉安发展的同志们、朋友们，致以衷心的感谢和崇高的敬意!

　　在肯定成绩的同时，我们也清醒认识到，我市经济社会发展中还存在不少矛盾和问题。综合实力仍然不强，产业规模不大、层次不高的问题仍较突出；创新能力较弱，人才支撑不足，体制机制障碍依然存在；部分群众生活还比较困难，与全国、全省同步全面小康任务还十分艰巨；政府公职人员的思想观念和能力水平，还不能完全适应新常态新要求，政风行风和廉政建设仍需加强，不作为、慢作为、乱作为现象仍有发生，个别公职人员甚至违法乱纪、贪污腐败。对此，我们一定高度重视并努力加以解决。

　　二、编制“十三五”规划纲要（草案）的主要考虑

　　“十三五”时期，是我市与全国全省同步全面建成小康社会的决胜阶段。市委统揽全局、集思广益，创造性地贯彻中央“四个全面”战略布局、“五大发展理念”，以及省委“十六字方针”，科学制定了“十三五”规划建议，成为引领吉安今后五年发展的行动纲领。市政府全面对标市委规划建议，组织编制了《吉安市国民经济和社会发展第十三个五年规划纲要》（草案），提交大会审议。

　　（一）关于“十三五”经济社会发展指导思想。指导思想是规划的灵魂。根据市委规划建议，《纲要》（草案）提出了未来五年发展的指导思想，重点强调了四个关键问题：一是强调“双中高”主线，即“经济增长中高速、产业迈向中高端”。提出“中高速”，主要是考虑到，发展不足、总量不大仍然是吉安的最大实际，低基数上的相对快增长仍然是我们发展的基本着眼点，只有保持较高增长速度，才能加快做大总量，加快追赶步伐。提出“中高端”，主要是考虑到，创新驱动、绿色发展是大势所趋、动力所在，只有推动产业向技术高端、产品革新、绿色环保升级，才能上规模、上能级，提升竞合力，赢得新优势。二是强调“两个立足点”，即“以提高发展质量效益和群众获得感为立足点”。这主要是坚持问题导向和民生导向相统一，既指明发展的前进方向与衡量标准，又阐明发展的出发点和落脚点。三是强调“两个动力”，即“以改革开放、创新创业为动力”。这主要是考虑，推动新一轮发展，必须充分发挥改革创新的先导作用，强化开明开放的引领作用，形成“大众创业、万众创新”的生动局面。四是强调“两个加快”，即“加快发展、加快脱贫”。这两者相辅相成、互促共进。吉安作为革命老区，脱贫任务十分艰巨，只有打赢加快发展、加快脱贫攻坚战，同步全面小康才有可能实现。

　　（二）关于“十三五”主要目标。根据市委规划建议，《纲要》（草案）从六个方面提出了未来五年预期目标，核心是“提前翻番、全面小康”，即与2010年相比，全市生产总值和城乡居民人均可支配收入提前实现翻一番，全面建成小康社会。围绕这一总目标，《纲要》（草案）从经济发展、转型升级、民生福祉、生态文明等方面，提出了36项具体目标。确定这些目标，考量的因素主要有三个方面：一是与同步全面小康相衔接。目前，吉安全面小康实现程度与全国全省平均水平相比还有较大差距，要实现同步全面小康，主要经济指标增速必须明显高于全国全省平均水平。为此，《纲要》（草案）设定我市生产总值年均增长10%左右，分别高于全国全省平均水平3.5和1.5个百分点。二是充分体现吉安特色和优势。《纲要》（草案）提出建设现代农业强市、旅游强市，打造吉泰走廊核心增长极、生态文明新样板、全国革命老区脱贫攻坚示范区、赣中区域性综合交通枢纽、江西重要的绿色能源基地等目标，都是在把握市情和优势基础上提出来的。三是兼顾必要性和可行性。从我市发展基础和潜力看，这些目标既鼓舞人心，又切实可行，只要加倍努力，完全可以实现。

　　（三）关于“十三五”重大战略任务。围绕实现五年目标，《纲要》（草案）对标市委规划建议，提出实施八大战略，即:实施吉泰走廊、脱贫攻坚、县域经济发展战略，构建协同梯度发展新格局；实施三产共生演进战略，构建更具竞争力的现代产业新体系；实施人才创新战略，培育经济社会发展新动力；实施开放引领战略，打造更宜投资创业新高地；实施吉泰城镇群与中小城镇协同发展战略，建设秀美宜居新家园；实施绿色崛起战略，打造处处绿水青山的生态文明新样板；实施共享发展战略，建设幸福新吉安；实施基础设施提升战略，构筑支撑经济社会发展的新优势。围绕八大战略任务，《纲要》（草案）提出全面深化改革、推进依法治市、加强和改善党的领导三大保障。这些战略任务和保障措施，是市委规划建议“八大战略、三大保障”的具体落实，既全面对接中央“五大发展理念”和省委“七大战略”，又与我市“十二五”发展相衔接，保持连续性，提高针对性，增强操作性。

　　各位代表，《“十三五”规划纲要》（草案）经大会讨论通过后，将成为我市“十三五”经济社会发展的宏伟蓝图，标志着我们开启建设开放繁荣秀美幸福新吉安的新征程。这是时代赋予我们的责任。只要我们同心同德，砥砺前行，同步全面小康的目标，就一定能够如期实现！

　　三、2016年工作安排

　　2016年是“十三五”开局之年，是全面建成小康社会决胜阶段的第一年，也是新常态下推进供给侧结构性改革輥輰訛的攻坚之年。今年经济下行压力仍将持续，同时也将迎来众多发展新机遇，我们将增强信心、保持定力，积极应对新挑战，迈出坚实新步伐。今年全市经济社会发展的总体要求是：深入贯彻落实党的十八大和十八届三中、四中、五中全会、中央经济工作会议，以及省委十三届十二次全会、全省经济工作会议、市委三届十一次全会、全市经济工作暨脱贫攻坚会议精神，主动适应新常态，树牢“五大发展理念”，紧扣省委“十六字方针”，把握稳中求进总基调，以推进经济增长中高速、产业迈向中高端为主线，以提高质量效益和群众获得感为立足点，大力实施五化同步互动、三产共生演进，强化改革攻坚，突出创新驱动，加快发展、加快脱贫，促进经济平稳较快发展，努力实现“十三五”良好开局，为“提前翻番、全面小康”打下坚实基础。

　　今年全市经济社会发展主要预期目标是：生产总值增长10%左右，财政总收入增长10%左右，规模工业增加值增长10%；固定资产投资增长15%以上，社会消费品零售总额增长12.5%以上，实际利用外资增长10%，外贸出口总额增长2%以上；城镇居民人均可支配收入增长9%，农村居民人均可支配收入增长10.5%左右，居民消费价格总水平涨幅控制在3%以内，城镇登记失业率控制在4.5%以内，节能减排完成省下达目标任务。

　　围绕上述目标要求，重点抓好以下八个方面的工作：

　　（一）扩大有效需求，促进经济增长“中高速”。充分发挥有效投资和企业帮扶的牵引作用，推动投资、消费、出口协同发力，持续增强经济增长的拉动力。

　　强化投资的关键作用。选准投资方向，重点围绕交通基础设施、产业发展、生态环保和社会事业等领域，组织实施400个以上增后劲、补短板、惠民生的重点项目。铁路方面，积极做好吉抚武、渝长厦项目前期工作，全力支持昌吉赣客专、蒙华煤运通道等项目建设；高速公路方面，实现东乡至昌傅高速吉安段全面竣工，力促广昌至吉安及吉安绕城项目顺利开工；航空航运方面，加快推进井冈山机场改扩建、新干航电枢纽等项目。拓宽项目筹资渠道，既充分发挥政府投资的杠杆效应，又强化社会投资的主体作用，有效开展股权、债券和PPP融资，力争年内发行企业债10亿元以上；同时，积极向上争资，紧盯国家政策动向和“三年投资滚动计划”，力促一批重大项目纳入国家和省“十三五”盘子，努力争取更多的中央和省预算内投资与专项建设资金，多渠道扩投资、稳增长。

　　发挥消费的基础作用。进一步拓宽消费领域，探索发行市内“旅游一卡通”，开通双休日城郊旅游公交，落实带薪休假制度，鼓励错峰休假，依法优化调整夏季作息安排，为职工周五下午与周末结合外出休闲度假创造有利条件；采取公建民营、民办公助、购买服务等多种形式，引导社会资本增加健康养老服务供给，拓展家政消费；落实小排量汽车、新能源汽车税费优惠政策，扩大汽车消费；打好房地产去库存歼灭战，推进农民工市民化，对首次购买城镇住房的符合条件的农民给予财政补贴，落实棚改货币化安置、保障房“以购以租代建”政策，培育公共住房租赁市场，激发住房刚性需求和改善性需求。同时，进一步改善消费环境，实施县乡农贸市场提升工程，开展“百城万店无假货”行动，切实维护消费者权益，让群众方便消费、放心消费。

　　巩固出口的促进作用。积极做大出口总量，全面落实鼓励出口的各项政策，力争发生出口实绩企业总数达到320户以上。不断做实出口平台，推动井冈山出口加工区申创综合保税区，加快完善吉安陆地港功能，提升进口商品直销中心运营水平。继续调优出口结构，开展生产型出口企业专项扶持行动，扩大机电和高新技术产品出口规模。

　　加大实体企业帮扶。开展“千组帮千企”活动，组织1000个以上工作组，帮扶1000户以上企业，扶大助小，扶优扶强。开展降低企业成本行动，落实税费减免政策，减轻“五险一金”负担，开通园区“微公交”，启动智慧园区建设，帮助企业降低人工、用电、用气和物流成本。健全政银企常态化对接机制，帮助企业解决“融资难、融资贵”问题。扩大帮扶覆盖面，优选一批有潜力、有市场的服务业和农业类企业纳入帮扶范围，实行“一企一策”、精准帮扶。

　　（二）实施创新驱动，推动产业迈向“中高端”。注重从供给侧发力，认真落实“去产能、去库存、去杠杆、降成本、补短板”五大任务，重点在创新创业、人才集聚方面下功夫，提高全要素配置效率，加快推动产业升级。

　　强化科技与人才创新。推进协同创新，策应全省“5511”工程輥輱訛，深化与高校院所、科研机构战略合作，新增2个省级工程技术研究中心，力争实现国家级创新平台零突破。强化企业自主创新，支持企业申报实施重大科技专项，新增省级企业技术中心3家，新增高新技术企业30户。推进“大众创业、万众创新”，加快创业孵化基地建设，设立创业引导基金，加大“创客”培育力度，建立两岸青年创新创业基地。加快补齐人才不足这块最大“短板”，全面落实吉安市“引才用才20条”，坚持刚性与柔性并举，开通引才“绿色通道”，加大科研奖励力度，新建一批人才公寓，创新方式，招引吉安籍人才回归创业，营造吸引人才、优待人才、用好人才的良好环境。

　　大力推进新型工业化。实施新一轮传统产业改造升级工程，推进燕京啤酒退城进园、井冈山卷烟厂易地改造项目竣工投产，推动钢铁、水泥、煤炭、建材等传统企业并购重组、转型升级，对资不抵债、扭亏无望的“僵尸企业”，妥善处置，平稳出清。加快壮大战略性新兴产业，深化“三年决战三个千亿”攻坚行动，力争全市战略性新兴产业主营业务收入突破1300亿元、占规模工业比重提高1个百分点。聚焦电子信息产业，实施“百亿企业培育计划”，制定落实LED和移动通讯终端两个千亿产业发展规划，建立协作联盟，开展专项对接，设立产业发展基金，组建LED国检中心，打造全省智能照明产业主要集聚区和移动通讯终端产业集聚区，力争电子信息产业主营业务收入超过680亿元。对接“中国制造2025”，促进新一代信息技术与数控机床、机电设备等产业深度融合，谋划通用航空产业园建设，打造先进装备制造业集聚区。推动园区转型升级，加快扩区调区，完善综合配套，新建标准厂房100万平方米。

　　加快发展现代农业。继续调优结构，在稳定粮食生产、畜禽水产养殖的同时，加快壮大六大富民产业，深入实施井冈蜜柚“千村万户”老乡工程，抓好苗木基地、有机肥厂、技术服务、销售体系建设，全市新增井冈蜜柚面积8万亩，新增绿色蔬菜、茶叶烟叶、高产油茶、珍贵楠木、竹木花卉面积27万亩。培育壮大新型经营主体，推动现代农业示范园扩容提质，力争核心区面积扩大到5万亩以上；推广“财政惠农信贷通”，加大对专业大户、家庭农场、农民合作社、龙头企业的金融扶持。推进农业品牌战略，严把品种品质关，扩大井冈蜜柚知名度，提升遂川狗牯脑茶、泰和乌鸡、安福火腿、永丰绿海茶油等传统品牌效益，唱响绿色有机品牌。推进农业“接二连三”工程，加快“一县一加工园”建设，大力发展休闲农业、电商农业。夯实农业基础，新建高标准基本农田12.3万亩，完成111座小（二）型以上病险水库除险加固，完善农机农技、气象水文、粮食收储、防灾减灾服务体系。

　　推动服务业提档升级。深入实施服务业发展提速三年行动计划，加快形成“一核一带两片区”輥輲訛格局，服务业占GDP比重超过35%。加快发展生产性服务业。完善物流园区平台，推进物流企业重组，积极发展“多式联运”，构建数字化、立体化物流体系；全面发展研发设计、检验检测、节能环保等新业态。大力提升生活性服务业品质。深化“三山一江一城”輥輳訛旅游发展战略，推进“大井冈”红色旅游一体化，支持青原山和吉州窑成功创建国家级风景名胜区，推动武功山全面融入“大武功全域旅游”，有序开发赣江水上旅游，推进古村古寺古书院旅游，打造环中心城区旅游圈，促进资源共享、互通联动；完善景区配套，基本完成旅游公厕建设；支持万安田北农民画村、泰和蜀口洲、永新三湾争创国家4A级景区，推动乡村旅游加快发展。推广品牌连锁经营模式，提升餐饮、商贸等传统服务业档次。乘势发展新兴服务业。实施“互联网+”行动，大力发展电子商务，建设8-10个特色电商产业园，推动电子商务进农村，争创国家电子商务示范城市。同时，统筹推进健康养老、文化创意、总部经济等各类业态竞相发展。

　　（三）聚焦重点战略，打造区域发展“增长极”。落实协调发展理念，坚定不移昂起龙头、挺起脊梁、立起支点，持续推进区域发展升级。

　　推进经开区和高新区联动发展。经开区高密度优化西区，高起点开发东区，高质量建设金鸡湖新区，完善孵化基地和医院学校等配套建设，抓紧组建企业家创业培训学院；加快产业集聚，启动吉安韩国产业园规划建设，推进木林森光电产业园、合力泰智能硬件及物联网产业园竣工投产，新增医药化工百亿产业集群，力争经开区规模工业主营业务收入超过580亿元，尽快进入全省园区十强。高新区聚焦高新技术产业，加快调规拓展，促进立讯产业园和聚能科技项目建设，力争规模工业主营业务收入超过350亿元。统筹经开区与高新区产业布局、优惠政策和投入强度，推动两个国家级园区互动融合、协调发展。

　　加快吉泰走廊率先发展。实施电子信息产业集聚工程，推进吉水军民结合示范产业园建设，启动井冈山机场临空经济区规划建设，力争走廊GDP和规模工业增加值分别达到750亿元和460亿元。继续推进走廊“六个一体化”，加快亚行贷款BRT项目建设，推动滨江大道北延，启动轻轨项目规划建设，实现新井冈山大桥、泰和大桥全面建成通车，完成客运一体化改造，推进中心城区到卫星镇、重点镇和工业园区公交全覆盖，促进基础设施和公共服务互联互通。加快吉泰城镇群建设，争取规划尽快获批，推进金樟、敦厚永和凤凰、澄江沿溪南溪和青原山四大组团建设，加速吉安县、吉水县融城步伐，全面构筑“大贯通、大组团”发展格局，成为推进苏区振兴、融入长江经济带的重要战略支点。

　　促进县域经济均衡发展。加大扩权强县力度，有针对性制定“一县一策”扶持措施，支持各地依托工业园区打造1-2个首位产业，力争吉州装备制造、泰和触控显示等产业集群过80亿元。做特城镇经济，推进扩权强镇试点扩面提质，引导产业基础较好、资源丰富的乡镇打造产业强镇，支持交通便利、人口集中的城关镇、中心镇打造商贸重镇，鼓励有文化底蕴、生态秀美的乡镇打造旅游名镇，形成“一镇一业”发展格局。落实户籍制度改革措施，实施居住证制度，促进产业向园区集中、人口向城镇集中，年内新增城镇户籍人口10万人以上。完善县域差异化考核，鼓励各地因地制宜打造产城融合示范区、生态文明示范区、扶贫攻坚示范区，力争所有县区财政总收入过10亿元。

　　（四）深化改革开放，培育经济发展“强引擎”。更加注重运用改革的办法、市场的力量，加快推动开放发展。

　　深化政府综合改革。着眼转职能、提效能，持续推进简政削权、放管结合、优化服务。加快行政审批制度改革，落实行政审批中介服务清单制度，完善行政权责清单动态调整机制，积极开展实效评估，确保下放事项落地落实。深化商事登记制度改革，实施“一址多照”和“一照多址”登记模式，探索企业简易注销改革。坚持放管结合，建立全市统一的监管平台和信用体系，健全信息披露和失信惩戒制度，做到“审批做减法、责任做加法、市场做乘法”。进一步优化服务，健全“一口式受理、一站式审批、一条龙服务”机制，推行并联审批、网上审批。深化财政体制改革，完善财政专项资金竞争性分配和项目化管理，实行政府债务余额限额管理，防范债务风险。

　　深化金融体制改革。完善金融体系，新引进一批股份制商业银行、证券公司、保险公司在吉安设立分支机构，吸引创投、风投等股权投资基金入驻吉安；推进民间资本管理公司、民间融资登记服务机构县域全覆盖。健全融资担保体系，推进融资担保机构增资扩股，组建市本级“续贷保”基金，实现倒贷基金县域全覆盖。创新金融产品和业态，发展互联网金融、普惠制金融，拓展县域金融、农村金融，年内金融机构新增贷款180亿元，存贷比提高3个百分点。推进多层次资本市场建设，推动建立现代企业制度，引导企业挂牌上市。优化金融生态，落实“去杠杆”任务，健全企业信用评价体系，打击非法融资，防范金融风险。

　　深化重点领域改革。继续聚焦“一廊四区”重点区域改革，深入推进吉泰走廊改革先行先试，落实工业园区规划和项目报批权限，深化景区“三权分离”改革。深化农业农村综合改革，巩固土地承包经营权确权登记颁证成果，加快农村综合产权交易市场建设，推进供销合作社和农垦系统改革发展。统筹推进机关事业单位养老保险等其他各项改革。

　　不断扩大对外开放。高水平“引进来”，强攻招商引资。强化重点区域招商，巩固长珠闽、突出港澳台、对接京津冀、拓展韩日欧，尤其要踩着不变的步伐，久久为功铆住对台招商，力争引进台资3亿美元，加快打造全省赣台产业合作示范区。拓宽招商领域，加大教育、文化、电商、旅游和农业全领域招商，每个县市区举办服务业专题招商活动1场以上。扭住招大引强，实施特大项目攻坚计划。创新招商方式，强化专业招商，开展以商招商，探索并购重组招商，推进“吉商回归工程”。同时，大力度“走出去”，设立“走出去”专项基金，支持名特优产品参加境外国外展销活动，推动优势产业参与国际合作。

　　（五）统筹城乡发展，打造城乡环境“升级版”。坚持精心规划、精致建设、精细管理，继续做好“减量、透绿、留白、传承、坚守”五篇文章，建设“见山见水，记得住乡愁”的宜居幸福家园。

　　聚焦庐陵新区和高铁新区建设。着力提升庐陵新区核心带动力，加快国贸中心、台商大厦等8个总部楼宇建设，推动铜锣湾、金鑫未来港二期等城市综合体招商开业，培育高端商贸、会展培训等城市业态，打造区域性城市CBD和全省产城融合示范区。重点推开城西高铁新区开发建设，高点定位，科学规划，年内做好10平方公里控制性详规，完成综合交通枢纽和站房的规划设计，加快土地收储和房屋征迁工作，启动西客站场、站前大道建设，为实现高铁新区“两年拉框架、打基础，三年通高铁、建新城”目标奠定基础。统筹推进中心城区“六大组团、八大片区”建设，拓展提升城东组团，加快把青原山打造成中心城区的后花园，推动城区由“单中心”向“多中心”转型，不断提高中心城区首位度。

　　持续提升城市功能和品位。紧扣民生需求，不断完善基础设施。按照“提升改建一批、布局新建一批、小区配建一批”的要求，大力推进菜市场建设，全市完成“农改超”30个以上，中心城区城南恒丰、恒大等菜市场建成投用。完善城市交通体系，大力实行公交优先，投放一批新能源优质公交车，规范出租车行业管理，在中心城区开工建设3个立体停车场，新增停车位3000个以上，有效解决停车难、打的难、出行难问题。加强供电供水、垃圾处理等设施建设，城北自来水厂年内建成投用，改扩建1-2个垃圾压缩中转站。深化“三改三清”攻坚行动，每个县市区打造3个以上示范片区。利用废旧厂房，改建一批居民健身娱乐活动场所，新建一批街头小游园。启动阳明西路地下综合管廊建设，实施污水管网改造工程，推进海绵城市建设。挖掘文化内涵，打造一批彰显庐陵风格的特色商业街区，实施田侯路庐陵文化古街区保护改造，提高中心城区庐陵商业街、仓口儒林里古街的经营管理水平。

　　强化城市精细化管理。推进智慧城市建设，加快打造“宽带吉安·光网城市”，中心城区年内实现光纤进小区全覆盖；实施一批基础和应用项目，加快社会服务信息化平台建设，实现线上线下互动，提高城市管理和服务的数字化、智能化、便利化水平。完善网格化管理，深化“人文社区·温馨家园”创建，提升物业管理水平。开展群众性精神文明创建活动，倡导崇德向善、遵德守礼浓厚氛围，促进社区共治与居民自治良性互动，争创全国文明城市。

　　丰富美丽乡村建设内涵。坚守村庄规划，推动镇村联动提档升级，完成第一批88个中心村建设，强化农民建房管控，加大“空心村”整治力度。深入实施农村清洁工程，推进农村生活污水治理，启动农村生活垃圾分类处理试点，实现中心村垃圾无害化、资源化处理全覆盖。加大传统民居、传统村落和少数民族特色村寨保护，打造一批庐陵文化特色名村。推行农村社区“131”治理模式輥輴訛，推进公共服务向农村延伸。实施新农民培训工程，推动“物的新农村”和“人的新农村”齐头并进。

　　（六）加快精准扶贫，建设老区脱贫“先行区”。立下愚公志，打一场提前脱贫攻坚战，确保年内再减贫10万人。

　　聚焦重点区域脱贫。以罗霄山片区为脱贫攻坚重点区域，以打通水电路、通信等基础设施“最后一公里”为目标，加大项目资金倾斜力度，年内实现25户以上自然村全部通水泥路，基本解决贫困村安全饮水问题。紧扣“提前脱贫”目标，加快5个国定贫困县脱贫步伐，年内吉安县、井冈山市实现脱贫摘帽。

　　创新精准脱贫举措。打好安居扶贫攻坚战，年内改造危旧土坯房6.1万户，基本完成改造任务，对确无能力改建的贫困户，实施“交钥匙”工程；对生产生活条件恶劣、不宜居住的地方，实施自然村整体移民搬迁，年内搬迁完成1.3万人。打好产业扶贫攻坚战，推行“龙头企业+基地+合作社+贫困户”、“致富能人+贫困户”等模式，引导贫困户因地制宜发展井冈蜜柚、绿色蔬菜、烟叶茶叶、毛竹加工等富民产业，增强贫困群众造血功能。打好保障扶贫攻坚战，加大医疗保险、社会救助保障力度，对丧失劳动能力的贫困人口进行兜底保障。强化贫困户主体意识，增强自主脱贫能力，不断提高脱贫质量，有效防止“被脱贫”。

　　构建社会化扶贫格局。落实扶贫责任，健全领导挂点和单位定点帮扶机制，继续选派干部驻村帮扶，每个乡镇成立扶贫工作站。加大扶贫投入，探索发行扶贫担保债，引导金融机构加大扶贫信贷投放。鼓励社会力量参与扶贫，开展村企共建、结对帮扶活动，建立健全激励机制，营造全社会关心扶贫、参与扶贫、支持扶贫的浓厚氛围。

　　（七）加强生态建设，构筑绿色崛起“新样板”。牢固树立绿色发展理念，增强保护生态就是厚植发展优势的坚定性，推进绿色惠民，增进生态福祉。

　　加强环境保护治理。坚持保护为先，严守“三条红线”，落实“四个一律不批”輥輵訛，绝不把降低环保门槛作为招商引资的优惠条件，绝不把严重污染项目引进吉安，绝不以牺牲环境为代价换取经济一时的发展。坚持整治为要，着力“净水”，全面推行“河长制”，落实治水净水责任；清理沿江、沿河污染企业，完善园区和城区污水处理设施，严格排污入河管理，确保达标排放；巩固水库退养成果，中小水库全面实行人放天养。着力“净土”，推进水土流失治理，做好生态修复工作，抓好土壤重金属污染治理。着力“净空”，实施蓝天行动计划，强化PM2.5实时监测，狠抓工业废气、城市扬尘和机动车尾气污染治理，加快淘汰黄标车和老旧机动车，保持好清新空气。

　　实施生态建养工程。提升通道绿化水平，广泛开展庭院绿化，推进植树造林、封山育林和低产低效林改造，新增造林38万亩，促进“道路林荫化、城市园林化、园区生态化、乡村林果化”。深化“两带一区”建设，加快赣江路堤结合项目建设，改造提升渼陂古村和东固景区，完成吉兴大道西延工程，推动赣江百里风光带尽快成势，促进都市田园休闲观光带全面成景、观光区基本成型。积极开展生态创建，争取永新、遂川、万安、安福列入国家重点生态功能区。加强湿地保护和修复，加快庐陵赣江和遂川五斗江两个国家湿地公园建设，推动万安湖创建国家湿地公园，尽快实现郊野公园县域全覆盖。

　　发展低碳循环经济。加快经开区国家循环化改造建设，争取吉安高新区、吉州工业园区省级循环化改造试点。推进“城市矿产”示范基地建设，支持永丰碳酸钙和安福铁尾矿资源综合利用创建国家“双百工程”示范基地。发展清洁能源，实施永丰灵华山、新干中电投等风电项目，加快吉泰走廊垃圾热电厂建设，推进光伏发电，打造江西重要的绿色能源基地。培育绿色生态文化，倡导低碳生活，引导绿色消费。

　　健全生态文明制度。探索生态补偿机制，积极争取江河流域生态补偿试点。认真落实环境保护“一岗双责”、自然资源离任审计制度和生态环境损害终身追究制，推动生态环保地方立法。加大环境执法力度，严惩违法违规行为，让破坏生态环境的“任性”行为付出沉重代价。

　　（八）强化民生保障，提升人民群众“获得感”。践行共享发展理念，切实办好民生实事，让发展成果更多惠及广大群众。

　　扎实推进民生工程。认真抓好省政府下达的50件民生实事和市民生工程。促进就业创业，组织实施大学生就业促进计划和创业引领计划，深入开展农民工就业培训行动，新增城镇就业4.5万人、农村劳动力转移就业6万人以上。健全社会保障体系，提高城乡低保、农村五保供养等社会救助标准，建立机关事业单位职业年金制度，健全基本医疗保险及大病保险、医疗救助制度，开展居家养老购买服务试点，全市223所敬老院、光荣院全部达到二星级以上标准。强化住房保障，新增保障性住房2450套，实施棚户区改造1.1万套。

　　加快发展社会事业。振兴发展教育事业。推进农村义务教育薄弱学校改造，新恢复一批村小和教学点，招聘乡村中小学教师1000名以上。优化城镇教育资源布局，有效化解“大班额”问题。抓紧解决幼师紧缺问题，年内75%以上乡镇中心幼儿园建成开学。积极推进双语学校建设。深化与深圳职业技术学院合作办学，规划建设金樟新区中职教育园区、经开区金鸡湖片区产城融合教育新区。大力建设健康吉安，健全医疗卫生服务体系，加强临床专科建设；推进市儿童医院、市中心医院内儿科医技大楼、井大附属医院综合大楼建设，将上海市东方医院吉安医院打造成赣中南区域医疗医学中心；加快建设吉安医学影像中心，探索建立分级诊疗制度；支持社会力量办医，推动医师多点执业；坚持计划生育基本国策，落实“全面两孩”政策，促进人口均衡发展；加强食品药品监管，保障人民群众身体健康和生命安全。繁荣文化体育事业，创建国家庐陵文化生态保护实验区；倡导全民阅读，打造“书香庐陵”；实施全民健身计划，实现市全民健身体育中心建成投用。

　　切实加强社会治理。推进法治吉安建设，建立健全公共法律服务体系，开展法律援助活动，实现法律顾问制度全覆盖；完善人民调解、行政调解、司法调解联动体系，落实“诉访分离”、“信访终结”制度，引导群众合法理性表达诉求。深化平安吉安建设，扎实做好安全生产工作，加大隐患排查与突出问题治理，守住安全底线，确保安全发展；认真做好民族宗教工作；深入开展“净安行动”輥輶訛，加强突发事件预警信息发布和应急处置工作，切实维护社会和谐稳定。

　　适应军队改革大势，加强国防动员和后备力量建设，深化军民融合，提升双拥创建水平。

　　各位代表，完成今年各项任务，实现“十三五”良好开局，对政府工作提出了新的更高要求。我们将大力践行“三严三实”要求，切实加强政府自身建设，不断提升工作水平。恪守为民施政，认真办理市长热线、市长信箱、政务微博、网上信访，及时回应群众期盼，切实做到重大决策为民而定、政策举措应民而出、安危冷暖唯民所系。推进依法行政，严格按照法定权限和程序履行政府职能，做到法定职责必须为、法无授权不可为；健全公众参与、专家论证、风险评估、合法性审查和集体讨论决定的行政决策机制，推进法治政府建设；支持人大地方立法和政协参政议政，自觉接受人大及其常委会的法律监督和政协的民主监督，认真办理人大代表建议和政协委员提案，广泛听取民主党派、工商联、无党派人士和各人民团体的意见。践行务实勤政，健全决策部署、监督反馈工作制度，加强部门协作和效能问责，大力整治懒政、怠政行为；完善重大项目领导挂点、重点工作现场办公、重要事项督促检查机制，反“四风”、改政风、树新风，把精力集中在“想干事”上，把本领展现在“会干事”上，把实效体现在“干成事”上，把底线设定在“不出事”上。坚持从严治政，自觉把纪律挺在前面，坚决贯彻中央“八项规定”精神以及省市有关规定，推动反腐倡廉向基层延伸，严肃查处发生在基层和群众身边的腐败行为；加强公共资源交易监管，加大行政监察和审计监督力度，立“明规矩”，破“潜规则”，扎牢“制度笼子”；落实公务用车制度改革，严格控制和压缩“三公”经费，把有限的财政资金用在刀刃上；加强对公职人员的教育、管理和监督，努力打造一支政治坚定、人民满意的政府公务员队伍。

　　各位代表！新的一年孕育新的希望，新的征途开启新的梦想。让我们更加紧密地团结在以习近平同志为总书记的党中央周围，在市委的坚强领导下，大力弘扬伟大的井冈山精神，团结拼搏、勇于担当，扎实苦干、创新求进，为建设开放繁荣秀美幸福新吉安、决胜同步全面小康而不懈奋斗!
